# A Method for Point Cloud Accuracy Analysis Based on Intensity Information

**DOI:** 10.3390/s23229135

**Published:** 2023-11-12

**Authors:** Siyuan Li, Dehua Zheng, Dongjie Yue, Chuang Hu, Xinjiang Ma

**Affiliations:** School of Earth Sciences and Engineering, Hohai University, Nanjing 211100, China; hhulsy@hhu.edu.cn (S.L.); 19950059@hhu.edu.cn (D.Z.); 201609020009@hhu.edu.cn (C.H.); mxj@hhu.edu.cn (X.M.)

**Keywords:** point cloud accuracy, precision analysis, intensity information

## Abstract

Three-dimensional laser scanning has emerged as a prevalent measurement method in numerous high-precision applications, and the precision of the obtained data is closely related to the intensity information. Comprehending the association between intensity and point cloud accuracy facilitates scanner performance assessment, optimization of data acquisition strategies, and evaluation of point cloud precision, thereby ensuring data reliability for high-precision applications. In this study, we investigated the correlation between point cloud accuracy and two distinct types of intensity information. In addition, we presented methods for assessing point cloud accuracy using these two forms of intensity information, along with their applicable scopes. By examining the percentage intensity, we analyzed the reflectance properties of the scanned object’s surface employing the Lambertian model. Our findings indicate that the Lambertian circle fitting radius is inversely correlated with the scanner’s ranging error at a constant scanning distance. Experimental outcomes substantiate that modifying the surface characteristics of the object enables the attainment of higher-precision point cloud data. By constructing a model associating the raw reflectance intensity with ranging errors, we developed a single-point error ellipsoid model to assess the accuracy of individual points within the point cloud. The experiments revealed that the ranging error model based on the raw intensity is solely applicable to point cloud data unaffected by specular reflectance properties. Moreover, the devised single-point error ellipsoid model accurately evaluates the measurement error of individual points. Both analytical methods can be utilized to evaluate the performance of the scanner as well as the accuracy of the acquired point cloud data, providing reliable data support for various high-precision applications.

## 1. Introduction

High-precision terrestrial laser scanning (TLS) has gained prominence in various fields due to its capability to efficiently and rapidly acquire precise 3D data over extensive areas. With the advancement and optimization of technology, acquiring high-precision data has become increasingly crucial in diverse fields such as precision mapping [1,2], engineering surveying [3], cultural heritage preservation [4,5], industrial manufacturing [6], mining exploration [7], transportation [8,9], and environmental monitoring [10]. In these domains, high-precision laser scanning data offer enhanced accuracy and reliability, thereby supporting decision making and practical applications.

However, the accuracy of point cloud data is subject to numerous factors, which can be classified into four primary categories:

1. Scanner mechanism: factors include the stability of laser emitters, performance of receivers [11], signal processing methods for distance measurement [12], angle measurement errors, axis alignment errors, etc;
2. Environmental factors: atmospheric conditions, lighting conditions, and the reflective properties of surrounding objects can influence laser propagation and consequently the accuracy of point cloud data;
3. Surface and scanning geometry: the color, texture, material, and roughness of an object’s surface, as well as the scanning distance and incident angle, can cause variations in the reflected laser intensity, impacting the ranging accuracy.
4. Scanner placement: unstable installation or imprecise positioning of the scanner can result in measurement errors.

To meet the high-precision requirements, researchers continuously strive to optimize laser scanning technology, enhancing data quality and application effectiveness. Among the four categories of errors previously mentioned, measurement errors arising from scanner mechanism can be eliminated or reduced through calibration methods [13,14]. With regard to instances of measuring real objects, measurement conditions are sometimes non-selectable; under such circumstances, the impact of environmental factors on point cloud accuracy needs to be studied under specific environmental conditions. When environmental conditions can be chosen, it is possible to select conditions with fewer micro-particles in the environment and no light for data collection, and to ensure accuracy and stability when placing the scanner, thereby enhancing the quality of the point cloud data. Due to the complexity and diversity of scanned targets, evaluating the accuracy of a specific category of scanned objects in practical application scenarios proves challenging. However, the intensity information is primarily influenced by surface properties and scanning geometry, which serves as a key parameter in the laser scanning process and significantly impacts point cloud accuracy.

Lichti et al. [15] investigated the impact of materials with varying surface reflectance properties on pulse laser ranging. Their study determined that the smoothness of the reflective surface influences the accuracy of laser pulse ranging. Additionally, materials with low reflectivity exhibit a shorter ranging distance compared to those with high reflectivity. Boehler et al. [16] carried out experimental tests on multiple laser scanners to evaluate their quality and measurement errors. Utilizing a range of test targets, such as planar objects with diverse reflectivities and white spheres, the researchers compared the measurement errors and point cloud noise of the scanners at varying distances and with different surface materials. This study presents a reliable method for assessing the performance of various laser scanners. Pfeifer et al. [17] performed a thorough analysis of the reflectance intensity information of a three-dimensional laser scanner (Riegl LMS-Z420i) and examined its effect on the scanner’s ranging outcomes. The experimental results suggested that the radiometric quality of the terrestrial laser scanner could be independently observed and analyzed. The authors advised against relying solely on the laser ranging equation to predict the intensity information provided by the scanner. Subsequently, the researchers examined the operational characteristics and reflectance information of two distinct laser scanners at varying distances and angles. They confirmed that the reflectance behavior of both scanners did not conform to the laser ranging equation [18]. Soudarissanane et al. [19] investigated the influence of scanning geometry factors on the signal-to-noise ratio of point clouds, and they developed a model to depict the relationship between local measurement noise, distance, and incidence angle. This model can be employed to optimize measurement settings and reduce measurement noise. Bolkas et al. [20] investigated the effects of scanning distance, incidence angle, target color, and glossiness on the quality of point clouds obtained by TLS. Their findings revealed that dark-colored targets exhibit higher point cloud noise compared to light-colored targets. Additionally, the researchers discovered that surface semi-glossiness can effectively decrease point cloud noise. This study offers valuable insights for users in selecting suitable scanners and enhancing the quality of TLS data acquisition. All of the aforementioned contributions hold substantial scientific significance, as they uncover various factors influencing the accuracy of 3D laser scanning point clouds. Nonetheless, developing a point cloud accuracy model that incorporates all of the previously mentioned influencing factors remains highly challenging. This is due to the variations in surface reflection characteristics of scanned objects, which necessitate distinct modeling. Wujanz et al. [21] investigated the impact of the interaction between the emitted signal and the object surface on ranging accuracy. They showed that the influence of various material targets on laser ranging can be represented by raw intensity values. Moreover, they developed a stochastic model to quantify the relationship between reflectance intensity information and ranging errors. To enhance the evaluation of point cloud accuracy, Chen et al. [22,23] incorporated the effects of angle measurement, range measurement, and laser beam spot into point cloud accuracy and devised an error ellipsoid model for assessing point cloud accuracy. Nevertheless, this model depended exclusively on measurement parameters supplied by the manufacturer, neglecting the impact of object surface material on ranging accuracy. Du et al. [24] and Ozendi et al. [25,26] also employed the error ellipsoid representation to describe the magnitude and direction of random errors for each data point. However, the source of ranging errors still did not account for the diversity and complexity of the scanned object surfaces.

As the utilization of TLS grows in fields demanding high precision, such as deformation monitoring and industrial manufacturing, a comprehensive understanding of 3D laser scanner performance is essential. To further enhance the quality of the acquired point cloud data and precisely assess the measurement uncertainty of the point cloud, we used the Zoller + Fröhlich Imager 5016 as an example to extensively examine the effect of intensity information on point cloud accuracy. We developed a single-point error ellipsoid model based on the raw intensity. Additionally, to address the issue of some scanners not providing access to raw intensity data, we employed the Lambertian reflectance model to examine the relationship between percentage intensity and ranging accuracy. The experimental findings can be applied to optimize scanner placement, enhance the accuracy of a collected point cloud, and accurately evaluate the spatial errors of point cloud data.

The organization of this paper is outlined as follows. Section 2 details the data acquisition scheme, the approach for analyzing percentage intensity, and the development of a single-point error ellipsoid using raw intensity. Section 3 presents the experimental findings obtained by analyzing the percentage intensity using the Lambertian reflectance model. Section 4 highlights the experimental outcomes of the single-point error ellipsoid model based on raw intensity. Lastly, Section 5 offers the conclusions and potential research directions for the proposed methodologies in this paper.

## 2. Materials and Methods

### 2.1. Sensor

The scanner used in this study was the Z + F Imager 5016, a compact and lightweight device designed for the rapid acquisition of high-resolution point cloud data. Table 1 displays the parameters provided by the manufacturer [27] for the Z + F Imager 5016, in which the measurement accuracy for horizontal and vertical angles is 0.004°. The linear error for distance measurement is less than 1 mm + 10 ppm/m, while the distance resolution is 0.1 mm.

As is stated in reference [21], the parameters of the random model, based on the raw intensity, fluctuate with the varying data rates of the scanner. Consequently, in this study, the point cloud density setting for all data acquisition processes was configured to ‘extremely high’, and the point cloud quality setting was adjusted to ‘premium’. Under this configuration, the data rate was 546.875 kHz.

### 2.2. Data Acquisition

**Figure 1:**
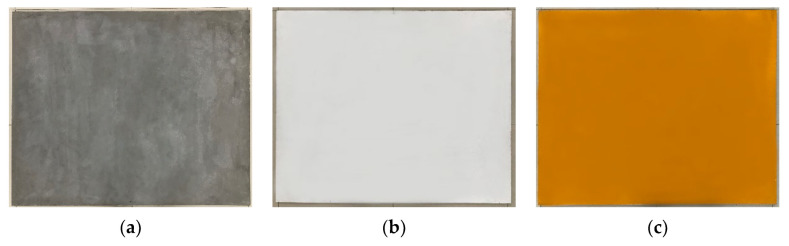
Tested object. (**a**) Concrete surface. (**b**) White painted surface. (**c**) Yellow painted surface.

To investigate the correlation between intensity and point cloud accuracy for various materials, this study employed three industrial wooden panels painted with a layer of concrete on their surfaces as scanning targets. The dimensions of these panels measured 1.2 m × 1.0 m. Two of the test panels featured a uniform application of white and yellow paint on the concrete surface, as depicted in Figure 1.

**Figure 2:**
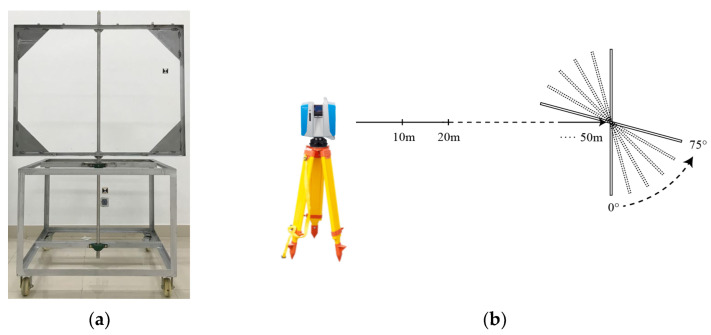
Workstation and data acquisition scheme. (**a**) Mobile workstation for placing tested object. (**b**) Data acquisition arrangements at different ranges and angles.

To facilitate the acquisition of point cloud data from the three panels at varying distances and angles, a mobile workstation was developed to house the panels, as shown in Figure 2a. The workstation guaranteed that the incident angle in the vertical direction remained close to zero during scanning. Moreover, the workstation incorporated a protractor to precisely adjust the horizontal angle of the panels, enabling alterations in the incident angle exclusively in the horizontal direction. This setup met the requirement of acquiring data at different incident angles.

The data acquisition scheme, illustrated in Figure 2b, employed scanning distances of 10 m, 20 m, 30 m, 40 m, and 50 m, collecting point cloud data at incident angles of 0°, 15°, 30°, 45°, 60°, and 75° for each distance. The experimental site consisted of an outdoor, open, and level road, and calibration was performed using a total station to guarantee the precision of scanning distances. The gathered point cloud data were converted to the ASCII format via the Z + F LaserControl (Version 9.3) software for further processing.

### 2.3. Plane Fitting and Residuals Analysis

The precision of the point cloud was determined by fitting the target point cloud data to a plane, followed by computing the root mean square error (RMSE) of the plane residuals. Plane fitting employs the method of total least squares, minimizing the orthogonal distance from the point to the fitted plane [28]. Before performing plane fitting, it is necessary to further segment the acquired point cloud data to avoid issues caused by the large area of the point cloud used for calculation at close distances, which may result in substantial differences in the incident angles of boundary points. Additionally, this step addresses potential minor impacts on the flatness of the experimental plate caused by vibrations generated from the workstation movement during the experiment. Using the centroid of each experimental panel’s point cloud as the center, a circular point cloud with a radius of 0.15 m was segmented for subsequent data analysis.

(1) $$a_{1}x+a_{2}y+a_{3}z+1=0$$

where $a_{1}$, $a_{2}$, and $a_{3}$ are the plane parameters. To further ascertain the suitability of the plane model for residual estimation, this study conducted a comparison between quadratic surface fitting and plane fitting results. The findings reveal that the residuals from quadratic surface fitting exceed those generated by plane fitting, thereby suggesting the plane model as a more appropriate option for error estimation.

**Figure 3:**
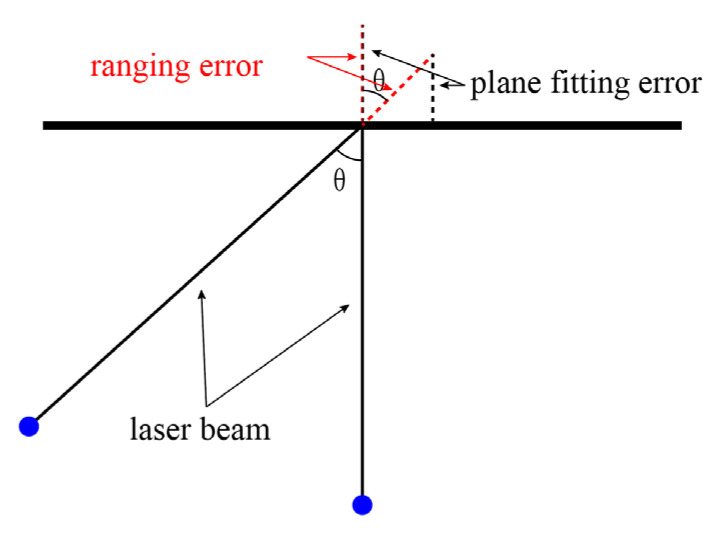
Plane fitting error and ranging error.

The calculation of plane fitting errors was obtained by computing the vertical distance from the points to the fitted plane, as shown in Figure 3. Due to the influence of the incident angle, a discrepancy exists between the plane fitting error and the ranging error as the incident angle gradually increases. The ranging error can be determined using the following equation:

(2) $$\rho_{r}=\frac{\rho_{p}}{\cos\theta }$$

where $\rho_{r}$ is the ranging error, $\rho_{p}$ is the plane fitting error, and θ is the incident angle.

### 2.4. Lambertian Reflectance Model

**Figure 4:**
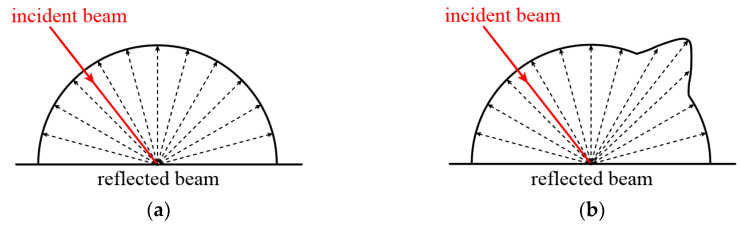
Light reflection model. (**a**) Ideal diffuse reflection model. (**b**) Combined specular and diffuse reflection model.

The Lambertian reflectance model is a classic model used to describe the reflection behavior of light on surface materials [29]. This model assumes that the surface is completely rough and uniform, neglecting effects like specular reflection and transmission. It is based on an idealized physical assumption that the intensity of the light reflected by the object’s surface remains consistent regardless of the observer’s viewing angle, resulting in a uniformly bright appearance, as illustrated in Figure 4a. Such ideal reflection conditions rarely exist in practical applications. Typically, the reflection of light on an object’s surface is a combination of diffuse reflection and specular reflection, as depicted in Figure 4b.

Point cloud data typically contain location information for each point as well as other related attributes, one of which is intensity. Intensity refers to the energy value of the light emitted by the LiDAR system when it strikes an object’s surface and reflects back. This intensity exhibits a direct proportionality to the power of the backscattered signal. Intensity values can provide information regarding the surface properties of an object, such as color, material, and roughness. The intensity recorded by scanners can be categorized into two types. The first category is the raw intensity, which corresponds to the intensity directly measured by the device. The measurement range may vary among different devices, and some manufacturers may not permit users to export these data. The second category is the normalized intensity, which standardizes the raw reflectance intensity values into a specific range, such as 0 to 1 or −1 to 1. For different scanners, the normalization methods for raw reflectance intensity may differ. For scanners that are unable to access raw intensity data, the normalized percentage intensity data and the Lambertian reflectance model can be utilized to analyze point cloud accuracy.

**Figure 5:**
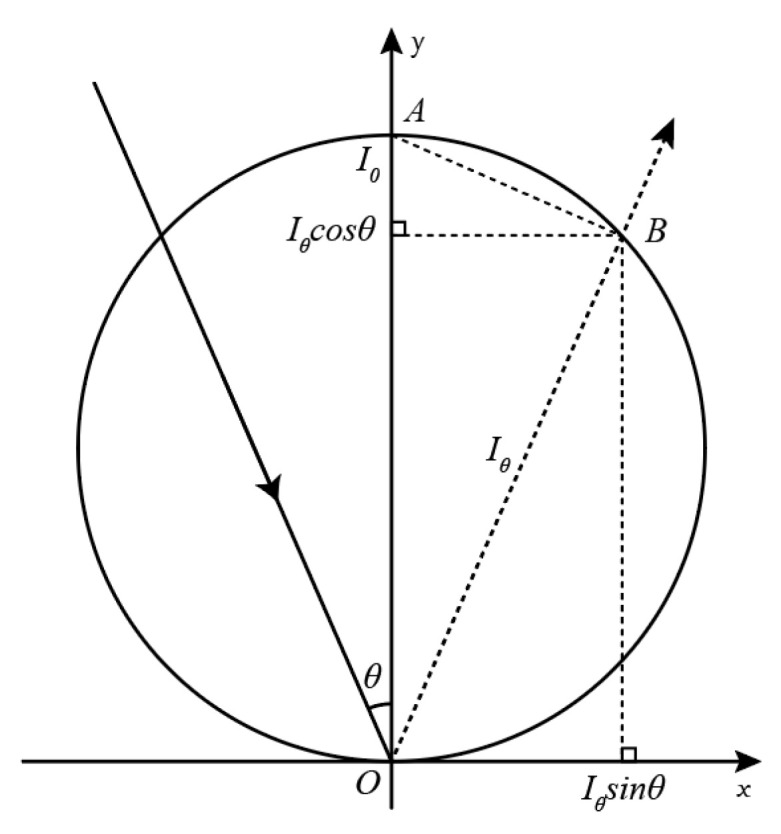
Lambertian circle.

The relationship between the radiant flux reflected within the solid angle and the radiant flux reflected in the surface normal direction for a Lambertian surface is as follows [30]:

(3) $$I_{\theta }=I_{0}\cos\theta$$

where θ denotes the angle between the incident direction and the surface normal; $I_{\theta }$ represents the radiant flux reflected in the specified direction within the solid angle, which can be expressed as the normalized intensity $R_{\theta }$; and $I_{0}$ is the radiant flux when the incident angle is 0°, i.e., a normalized intensity $R_{0}=1$. The relationship between the reflectance intensity of a Lambertian surface and the incident angle can be described using a Lambertian circle, as shown in Figure 5.

In Figure 5, *OA* represents the reflectance intensity $I_{0}$ when the incident angle is 0°, and *OB* represents the reflectance intensity $I_{\theta }$ when the incident angle is θ. The reflectance intensity and the incident angle follow the parametric equation below:

(4) $$\left\{\begin{array}{l} x_{r}=I_{\theta }\sin\theta \\ y_{r}=I_{\theta }\cos\theta \end{array}\right.$$

The $x_{r}$ and $y_{r}$ of each data group at the same distance can be calculated based on the incident angle and the mean percentage intensity of the test panel. The $x_{r}$ and $y_{r}$ of data with different incident angles at the same distance can be fitted to a Lambertian circle using the model in Equation (5):

(5) $$x_{r}^{2}+y_{r}^{2}+b_{1}x_{r}+b_{2}y_{r}+b_{3}=0$$

where $b_{1}$, $b_{2}$, and $b_{3}$ are the parameters of the Lambertian circle. The Lambertian circle radius can be used to analyze the Lambertian characteristics of different materials and serve as a basis for evaluating the scanning data accuracy of various materials.

### 2.5. Single-Point Error Ellipsoid Model

The error ellipsoid is a geometric shape used to describe the uncertainty of a position or state. When a scanner records the coordinates of a point, there will always be errors caused by distance and angle measurements. By utilizing the error ellipsoid, the uncertainty in spatial location of a point caused by these errors can be effectively described. For any point in Cartesian space, its spatial uncertainty can be represented as Equation (6):

(6) $$f(x,y,z)=\frac{1}{{\left(2\pi \right)}^{3/2}\sqrt{\left|D_{x,y,z}\right|}}\exp(-\frac{1}{2}R^{T}D_{x,y,z}^{-1}R)$$

where $R={\left[x-x^{*},y-y^{*},z-z^{*}\right]}^{T}$ represents the difference between the true value and the measured value of a point, while $D_{x,y,z}$ is the covariance matrix.

When a scanner records the coordinates of a point, it usually uses polar coordinates. During subsequent processing, the polar coordinate system is converted into the Cartesian coordinate system:

(7) $$\left\{\begin{array}{l} x=\rho \sin\alpha \cos\beta \\ y=\rho \sin\alpha \sin\beta \\ z=\rho \cos\alpha \end{array}\right.$$

In this equation, *x*, *y*, and *z* are the Cartesian coordinates of a point, ρ is the range measured by the scanner, α is the vertical angle, and β is the horizontal angle.

Without considering the influence of atmospheric and other factors, the uncertainty of a point is mainly affected by distance measurement and angle measurement, and follows a normal distribution. Based on the error propagation law, the coefficient propagation matrix **K** can be obtained by taking the total differential of Equation (7).

(8) $$K=\left[\begin{array}{ccc} \sin\alpha \cos\beta & \rho \cos\alpha \cos\beta & -\rho \sin\alpha \sin\beta \\ \sin\alpha \sin\beta & \rho \cos\alpha \sin\beta & \rho \sin\alpha \cos\beta \\ \cos\alpha & -\rho \sin\alpha & 0 \end{array}\right]$$

The range and angle measurements of the scanner are uncorrelated; thus, the covariance matrix $D_{\rho ,\alpha ,\beta }$ for range and angle measurements is

(9) $$D_{\rho ,\alpha ,\beta }=\left[\begin{array}{ccc} \sigma_{\rho }^{2} & 0 & 0\\ 0 & \sigma_{\alpha }^{2} & 0\\ 0 & 0 & \sigma_{\beta }^{2} \end{array}\right]$$

where $\sigma_{\rho }$ represents the ranging error, $\sigma_{\alpha }$ represents the vertical angle error, and $\sigma_{\beta }$ represents the horizontal angle error. Based on the error propagation law and the coefficient matrix **K**, the covariance matrix of the point’s coordinates in the Cartesian coordinate system can be calculated:

(10) $$D_{x,y,z}=KD_{\rho ,\alpha ,\beta }K^{T}=\left[\begin{array}{ccc} \sigma_{x}^{2} & \sigma_{xy} & \sigma_{xz}\\ \sigma_{xy} & \sigma_{y}^{2} & \sigma_{yz}\\ \sigma_{xz} & \sigma_{yz} & \sigma_{z}^{2} \end{array}\right]$$

Hence, the error ellipsoid for a point in the Cartesian coordinate system can be described as

(11) $$R^{T}D_{x,y,z}^{-1}R=k^{2}$$

where *k* is the amplification coefficient of the error ellipsoid, determined by a given probability. The covariance matrix $D_{x,y,z}$ can be converted into a diagonal matrix using an orthogonal matrix **Q**. Then, Equation (11) can be converted into

(12) $$R^{T}D_{x,y,z}^{-1}R=R^{T}Q^{T}\Lambda^{-1}QR={\left[\begin{array}{l} u\\ v\\ w \end{array}\right]}^{T}\left[\begin{array}{ccc} \lambda_{1}^{-1} & & \\ & \lambda_{2}^{-1} & \\ & & \lambda_{3}^{-1} \end{array}\right]\left[\begin{array}{l} u\\ v\\ w \end{array}\right]=k^{2}$$

In this equation, $\lambda_{1}$, $\lambda_{2}$, and $\lambda_{3}$ are the three eigenvalues of the covariance matrix $D_{x,y,z}$. Equation (12) can be described as the relationship between the error ellipsoid and the covariance matrix, and it can be rewritten as a standard ellipsoid equation:

(13) $$\frac{u^{2}}{\lambda_{1}^{}}+\frac{v^{2}}{\lambda_{2}^{}}+\frac{w^{2}}{\lambda_{3}^{}}=k^{2}$$

In most instances, the scanner’s angle error can be derived from values provided by the manufacturer or calculated following scanner calibration. The ranging error provided by the scanner manufacturer is a rather general description, but there is a specific functional relationship between ranging error and the raw intensity. By constructing a random model for raw intensity and ranging error, the ranging error can be determined through the raw intensity value.

(14) $$\sigma_{\rho }=c_{1}\cdot Rf^{c_{2}}+c_{3}\cdot Rf^{c_{4}}$$

where *Rf* denotes the raw intensity, and $c_{1}$, $c_{2}$, $c_{3}$, and $c_{4}$ are fitting parameters. Utilizing this random model, the spatial error ellipsoid of any point within the point cloud can be computed based on the raw intensity data and angle error. In actual applications, error values in arbitrary directions can be determined according to the point’s error ellipsoid.

## 3. Accuracy Analysis Based on Percentage Intensity

**Figure 6:**
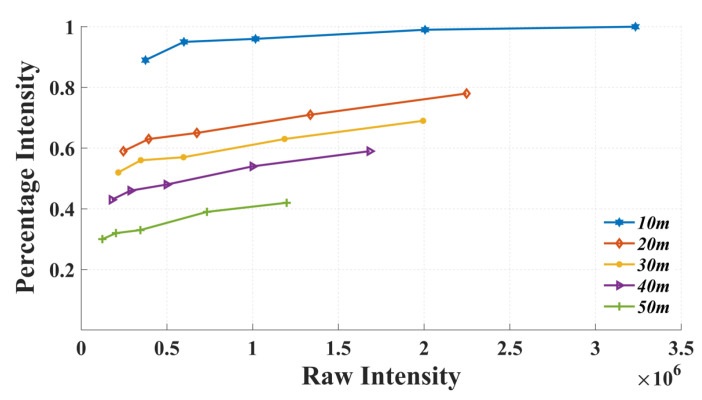
The relationship between raw intensity and percentage intensity.

During the experimental data collection with the Z + F Imager 5016, both percentage and raw intensities for each point were concurrently documented. It became clear that the scanner’s recorded percentage intensity is more than a simple normalization of the raw intensity. As is illustrated in Figure 6, at the same range and due to variations in incident angles, both reflection intensities exhibit a positive linear relationship. This relationship remains consistent when the range changes. However, at varying distances, the same raw intensity value may correspond to multiple percentage intensities. It can be deduced that the percentage intensity recorded by the Z + F Imager 5016 is a function of both raw intensity and range. As a result, subsequent analyses focused solely on comparing values at the same range.

### 3.1. Lambertian Circle Fitting and Analysis

**Figure 7:**
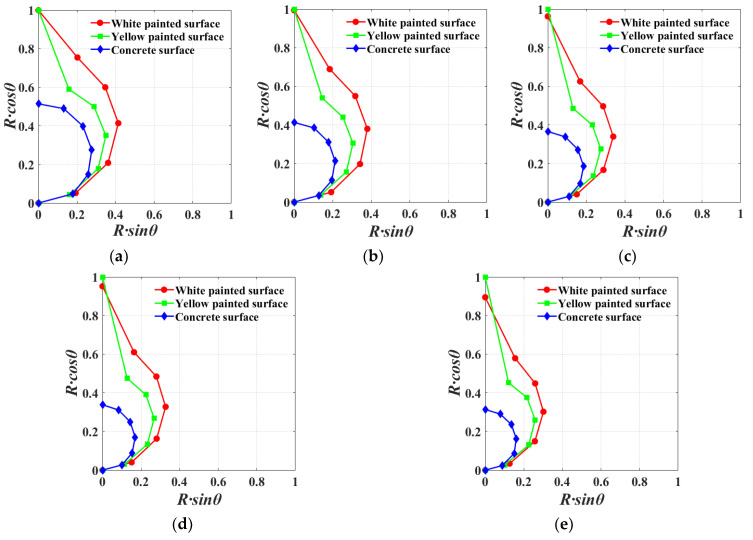
Correspondence between average intensity and incident angle at (**a**) 10 m, (**b**) 20 m, (**c**) 30 m, (**d**) 40 m, and (**e**) 50 m.

Using the experimental plan introduced in Section 2, point cloud data were acquired at various distances for the concrete surface, white painted surface, and yellow painted surface, for each distance, with 15° intervals ranging from 0° to 75°. Employing the mean percentage intensity and incident angle for each point cloud dataset, $x_{r}$ and $y_{r}$ were calculated using Equation (4), revealing the relationship between the average intensity and incident angle for the experimental panels, as illustrated in Figure 7.

**Figure 8:**
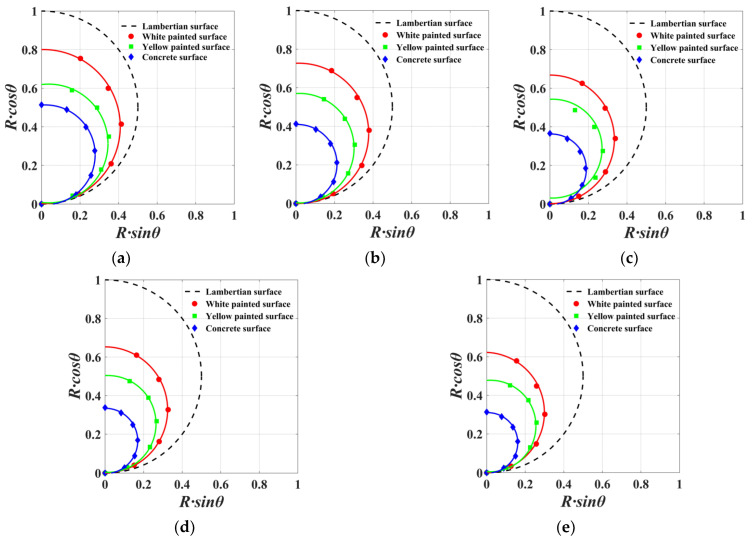
Lambertian circle fitting results at different ranges: (**a**) 10 m, (**b**) 20 m, (**c**) 30 m, (**d**) 40 m, and (**e**) 50 m.

As is demonstrated in Figure 7, the intensity of both the white and yellow painted surfaces approached 100% at a 0° incident angle, indicating that these materials predominantly exhibit specular reflection characteristics at this angle. When the incident angle surpasses 0°, the curve depicting the relationship between intensity and incident angle conforms to Lambertian properties. Thus, white and yellow painted surfaces exemplify typical mixtures of specular and diffuse reflections. A concrete surface displays less prominent specular reflection features at a 0° incident angle, more closely aligning with Lambertian characteristics. To further ascertain the Lambertian circle parameters for the three materials, the fitting model from Equation (5) was employed to analyze the data. Due to the presence of specular reflection features in the white and yellow painted surfaces at 0°, only data from incident angles between 15° and 75° were used for fitting these materials. The results are shown in Figure 8.

**Figure 9:**
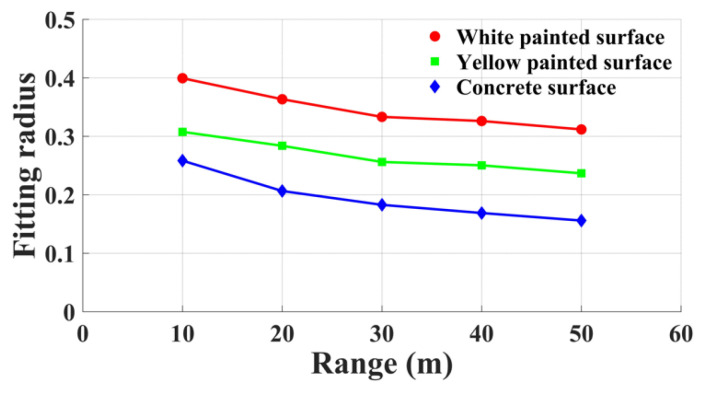
Variations in the Lambertian circle radius as a function of range.

**Figure 10:**
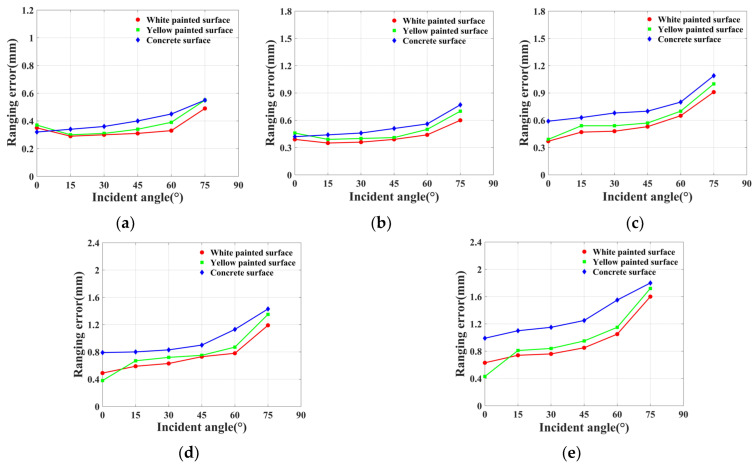
Correlation between incident angle and ranging error at different ranges: (**a**) 10 m, (**b**) 20 m, (**c**) 30 m, (**d**) 40 m, and (**e**) 50 m.

The Lambertian circle fitting results indicate that, in the absence of considering the vertical incidence of the laser beam, the experimental panels for all three materials conform to Lambertian properties. The white painted surface displays the largest Lambertian circle fitting radius, while the concrete surface exhibits the smallest. Additionally, the Lambertian circle fitting radius decreases progressively as the scanning distance expands. Figure 9 presents the trends in terms of changes in the Lambertian circle radii for the three materials within a scanning distance range of 10 m to 50 m. Figure 10 depicts the variations in ranging errors for the three materials across different angles and distances.

By combining Figure 9 and Figure 10, it becomes evident that, at a fixed scanning distance, there is a negative correlation between the Lambertian circle fitting radius and ranging error. Consequently, a larger Lambertian circle fitting radius leads to higher point cloud accuracy. Scanning ranges of 10 m and 20 m reveal that white and yellow painted surfaces exhibit higher ranging errors at 0° incident angles compared with 15° incident angles, due to accuracy losses caused by specular reflection effects at 0°. When the energy of the reflected beam received by the scanner surpasses a specific threshold, the ranging accuracy declines, which is generated from the scanner. At a scanning range of 30 m, the aforementioned phenomenon is absent for the white painted surface, as the laser beam expends some energy during its outbound and return paths, causing the energy of the received beam to fall below the threshold. For scanning distances of 40 m and 50 m, the yellow painted surface demonstrates a lower ranging error at a 0° incident angle than the white painted surface, which is attributable to its stronger specular reflection characteristics. When the incident angle surpasses 15°, the white painted surface’s ranging error becomes lower than that of the yellow painted surface, as a result of the yellow painted surface’s more pronounced specular reflection characteristics. As the incident angle increases, the majority of the energy in the laser beam emitted by the scanner is influenced by specular reflection and cannot be received by the scanner. In summary, the white painted surface displays the lowest ranging error, whereas the concrete surface exhibits the highest ranging error.

This finding highlights the significant impact of the returned beam’s energy magnitude on point cloud accuracy for laser scanners. In order to obtain point cloud data that satisfies the accuracy requirements of specific applications, a comprehensive evaluation of the scanner’s performance is crucial. By analyzing the scanner’s ranging error at different ranges and incident angles, a preliminary understanding of scanner performance can be obtained.

By analyzing the ranging errors of the scanner at various distances and incident angles, a preliminary understanding of the scanner’s performance can be achieved, determining whether the scanning accuracy of the scanner can meet the precision requirements of meticulous applications.

Moreover, in some precision applications, such as deformation monitoring, if circumstances allow, the reflective properties of the surface of the object being scanned can be altered by applying an appropriate coating. Through this approach, the Lambertian characteristics of the scanned object’s surface are enhanced, and the ability of the object’s surface to reflect light beams through diffuse reflection is strengthened, thereby enabling the acquisition of high-quality, high-precision point cloud data.

### 3.2. Reflectance Enhancement Experiment

**Figure 11:**
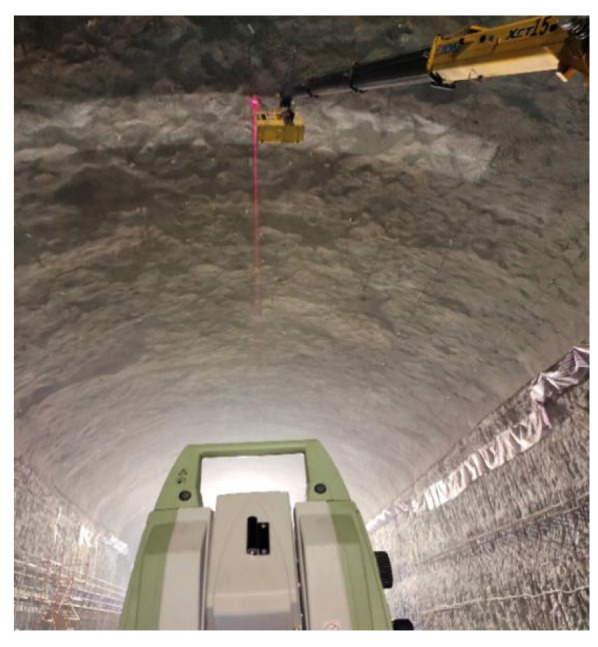
Cavern with a part of the roof painted white.

High-precision point cloud data are crucial for a variety of precision applications in 3D laser scanning technology. To obtain such data, it is essential to understand the performance of the scanner in use and to scientifically set up the scanner based on its capabilities. Moreover, modifying the reflective properties of the scanned object’s surface can enhance the energy of the returned beam received by the scanner. To validate the conclusion that altering surface characteristics can yield higher-precision data, we compared the registration errors of point clouds from the concrete surface and the white painted surface in two different periods using data from a cavern of the main underground plant of a pumped storage power station with a part of the roof painted white (Figure 11). The two sets of data were collected with a one-month interval, and the selected unpainted and painted concrete surfaces were located on either side of a cross-section within the cavern. This cross-section was outfitted with strain gauges and a total station monitoring prism. Upon analyzing the data from the strain gauges and total station, the impact of cavern deformation on the registration results was ruled out. During data collection, the scanner was placed directly beneath the midpoint between the two experimental areas, ensuring equal average distances from the scanner to both regions.

**Figure 12:**
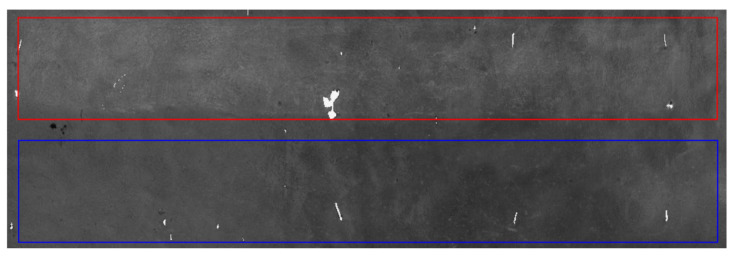
Experimental area. Red frame indicates white painted surface; blue frame indicates concrete surface.

The first point cloud dataset that was collected is shown in Figure 12. It is evident from the point cloud that the intensity of the white painted surface is higher than that of the concrete surface. The average intensity values for the concrete and white painted surface in both datasets are presented in Table 2.

**Figure 13:**
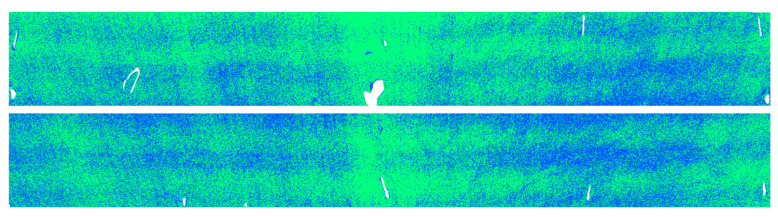
Registration result.

To mitigate the impact of the points representing the anchor rods and the total station prism on registration accuracy, manual removal of these points was implemented in both datasets. Point clouds from the experimental areas in the two periods were individually cropped and registered. Coarse registration utilized pre-positioned target spheres within the cavern, while the ICP algorithm was employed for precise registration. Figure 13 displays the registration results, and Table 3 presents the registration errors.

Under identical scanning conditions, higher point cloud data accuracy corresponds to thinner data layers, which then results in lower root-mean-square errors after registration. The experimental findings reveal that painting the cavern vault’s concrete surface white can elevate data intensity by approximately 20%, consequently improving point cloud accuracy. Considering the one-month interval between the two data collection periods, dust and oil fumes accumulated on the vault surface, diminishing the intensity of the second-phase dataset compared to the first. Nonetheless, the white painted surface in the second-phase exhibited a higher intensity than the concrete surface point cloud data in the first phase.

## 4. Error Ellipsoid Model for Single-Point Accuracy Estimation

Acquiring high-precision point cloud data is crucial for precision applications, and accurate assessment of the data accuracy is of significant importance in such applications, such as model reconstruction and deformation analysis, among others. A rational evaluation of point cloud accuracy aids in comprehending data quality and can function as a quality control measure. By examining point cloud accuracy, potential issues within the dataset, such as whether the data precision meets the application requirements, can be detected, thereby informing decision-making processes.

### 4.1. Model of Ranging Accuracy Based on Raw Intensity

In the data collection experiment outlined in Section 2, 90 sets of point cloud data were gathered, encompassing various angles, ranges, and surface materials. Within these sets, the data for white and yellow painted surfaces at a 0° incident angle were influenced by specular reflection properties, resulting in elevated ranging errors. Furthermore, under such conditions, the scanner registered abnormally high raw intensity, deviating from the random model presented in Equation (14). Consequently, data impacted by specular reflection properties were excluded from random model fitting. As was deduced in Section 3, the experimental data at a 0° incident angle, for white painted surfaces at scanning distances of 10 m and 20 m, as well as yellow painted surfaces at scanning distances of 10 m, 20 m, and 30 m were omitted from the random model fitting.

**Figure 14:**
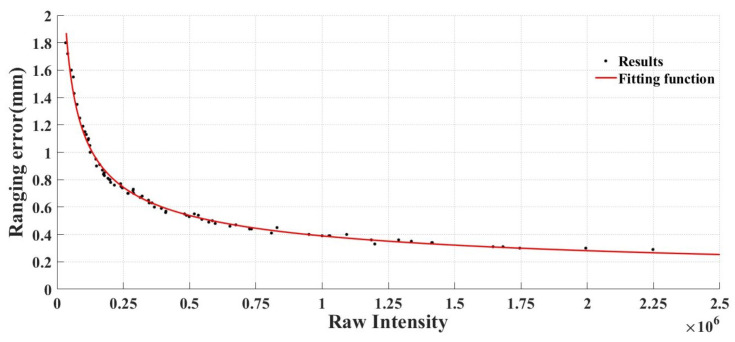
Fitting result.

Based on the experimental data, Figure 14 shows the random model fitting results, while Table 4 provides the corresponding fitting parameters.

Figure 13 and Table 4 demonstrate that the overall random model fitting results are satisfactory. It is crucial to acknowledge that various scanners produce different random model parameters at distinct data rates, and corresponding random models should be established according to specific data rates of different scanners. Furthermore, this random model is unable to precisely evaluate the ranging error of point cloud data exhibiting strong specular reflection properties at a 0° incident angle, although such situations are relatively rare in practical applications. When employing this random model, special consideration should be given to point cloud data at a 0° incident angle. In light of the actual conditions and in combination with the analytical methods presented in Section 3, the existence of any specular reflection properties can be ascertained. If such properties are detected, the point cloud accuracy can be evaluated through further experiments based on the scanning range of these points, or by altering the surface properties of the scanned objects to improve or eliminate the impact of specular reflection on the random model.

### 4.2. Experiment for Evaluating Point Cloud Accuracy

**Figure 15:**
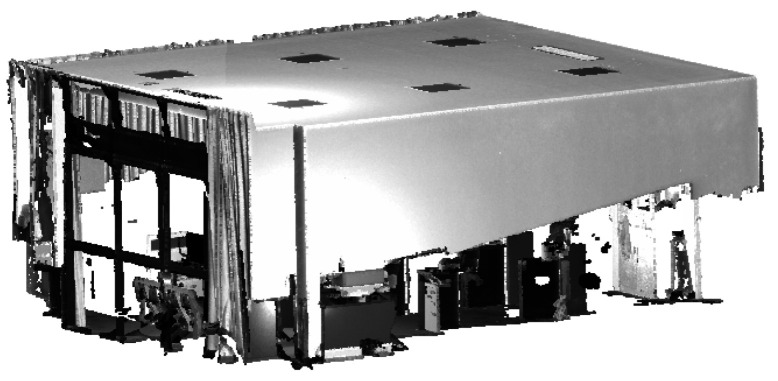
Point cloud for an office.

To confirm the single-point error ellipsoid model’s ability to accurately evaluate point cloud data accuracy, point cloud data from an office was gathered using a consistent scanner and sampling settings, as depicted in Figure 15. The planar characteristics of the ceiling, walls, and floor were employed to compute the root-mean-square error and maximum error for plane fitting. Concurrently, the single-point error ellipsoid model was applied to estimate the average maximum error for each point along the direction of the plane normal vector. The accuracy of the model was validated by comparison.

**Figure 16:**
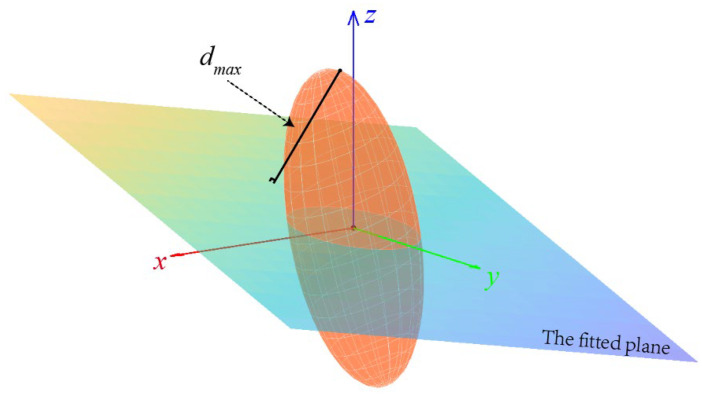
Error ellipsoid and fitted plane.

When employing the error ellipsoid to determine the plane fitting error of an individual point, the assumption is that the point’s true value resides on the fitted plane. The fitted plane’s normalized normal vector is represented by $n_{p}={\left(n_{1},n_{2},n_{3}\right)}^{T}$. To facilitate computation, both the point and the fitted plane can be translated to the origin, as depicted in Figure 16.

Calculate the distance, *d*, from the points on the error ellipsoid to the fitted plane:

(15) $$d=\left|x\cdot n_{1}+y\cdot n_{2}+z\cdot n_{3}\right|$$

The maximum distance, $d_{\max}$, from the points on the error ellipsoid to the fitted plane can be determined using Lagrange optimization, subject to the constraint:

(16) $$R^{T}Q^{T}\Lambda^{-1}QR-k^{2}\leq 0$$

The scanner’s ranging error is derived from the random model presented in Section 4.1, while the angle error is directly taken as $\sigma_{\alpha }=\sigma_{\beta }=0.004^{\circ }$, as stated in the scanner’s manual. It is necessary to convert this value to radians for calculation. Within the office point cloud, five datasets were selected, targeting the ground, walls, and ceiling. The corresponding RMSE and maximum error for plane fitting, and error estimates for these points were computed, with the results presented in Table 5.

As can be observed in Table 5, the maximum error values and those estimated by the error ellipsoid model exhibit close proximity within each dataset. Notably, the error assessments derived from the error ellipsoid slightly exceed the actual measured values, as the model incorporates extreme cases. Consequently, the error ellipsoid model, grounded in the original reflected intensity and proposed in this study, offers an accurate and reliable means of assessing point cloud accuracy.

## 5. Conclusions

This study analyzed the relationship between two types of reflected intensity and point cloud accuracy, using data obtained from the Z + F Imager 5016 3D laser scanner. A point cloud accuracy analysis method based on the Lambertian reflectance model and per-centage intensity was proposed, which is applicable for assessing the performance of the scanner and the accuracy of the acquired point cloud data in situations where raw intensity information is unavailable. On this basis, the impact of enhancing surface reflective characteristics on point cloud accuracy was verified. The results indicate that altering the reflective properties of an object’s surface can effectively increase the percentage of reflective intensity values obtained by the instrument while maintaining the Lambertian characteristics of the scanned object’s surface, thereby enhancing the overall accuracy of the point cloud data. This outcome can be employed to acquire high-precision point cloud data in applications such as deformation monitoring or precise modeling.

The study also analyzed the raw intensity obtained by the scanner. An error model of ranging was established based on the raw intensity. The experimental results indicate that the model is applicable to laser echoes from non-specular reflection received by the scanner. When the incident angle is 0°, the Lambertian model can be used to determine whether there is specular reflection characteristic. For such data, further experiments need to be set up to analyze the accuracy of point clouds. Furthermore, a single-point error ellipsoid model based on the raw intensity is proposed for assessing the quality of the point cloud. By utilizing a spatial plane model, it has been verified that the error ellipsoid model with *k* = 1 can accurately predict the measurement errors of the point cloud data.

In subsequent work, the two analysis methods discussed above will be further refined to develop a ranging accuracy model based on the raw intensity. This model will incorporate segmented functions to represent ranging errors associated with specular reflection and diffuse reflection characteristics. Additionally, in the single point error ellipsoid model, other potential influencing factors will be introduced, such as the impact of the laser spot on the uncertainty of the point cloud.

## Figures and Tables

**Table 1:** Technical specifications.

Elements	Scan Scope	Resolution	Accuracy
Range	0.3~365 m	0.1 mm	≤1 mm + 10 ppm/m
Vertical	320°	0.00026°	0.004°
Horizontal	360°	0.00018°	0.004°

**Table 2:** The intensity values for the two types of surface across both datasets.

Test Area	Average Intensity	Improvement (%)
First-Phase Concrete Surface	0.32	-
First-Phase White Painted Surface	0.39	22%
Second-Phase Concrete Surface	0.29	-
Second-Phase White Painted Surface	0.36	24%

**Table 3:** Registration errors.

Test Area	RMSE (mm)	Improvement (%)
Concrete Surface	1.0	-
White Painted Surface	0.8	20%

**Table 4:** Fitting parameters.

Model Parameters				RMSE (mm)	R-Squared
*c*_1_	*c*_2_	*c*_3_	*c*_4_		
253.8	−0.4694	6.416	−8.354	0.03	0.99

**Table 5:** The calculation results of each dataset.

Test Area	RMSE (mm)	Max Error (mm)	Evaluated Error (*k* = 1) (mm)
Ground	0.20	0.72	0.85
Wall 1	0.12	0.35	0.36
Wall 2	0.15	0.45	0.47
Ceiling 1	0.13	0.37	0.40
Ceiling 2	0.14	0.43	0.43

## Data Availability

The data presented in this study are available in insert article.
